# Threshold Levels of Infant and Under-Five Mortality for Crossover between Life Expectancies at Ages Zero, One and Five in India: A Decomposition Analysis

**DOI:** 10.1371/journal.pone.0143764

**Published:** 2015-12-18

**Authors:** Manisha Dubey, Usha Ram, Faujdar Ram

**Affiliations:** 1 International Institute for Population Sciences (IIPS), Mumbai, Maharashtra, India; 2 Department of Public Health and Mortality Studies, International Institute for Population Sciences (IIPS), Mumbai, Maharashtra, India; Institute for Health & the Environment, UNITED STATES

## Abstract

**Objectives:**

Under the prevailing conditions of imbalanced life table and historic gender discrimination in India, our study examines crossover between life expectancies at ages zero, one and five years for India and quantifies the relative share of infant and under-five mortality towards this crossover.

**Methods:**

We estimate threshold levels of infant and under-five mortality required for crossover using age specific death rates during 1981–2009 for 16 Indian states by sex (comprising of India’s 90% population in 2011). Kitagawa decomposition equations were used to analyse relative share of infant and under-five mortality towards crossover.

**Findings:**

India experienced crossover between life expectancies at ages zero and five in 2004 for menand in 2009 for women; eleven and nine Indian states have experienced this crossover for men and women, respectively. Men usually experienced crossover four years earlier than the women. Improvements in mortality below ages five have mostly contributed towards this crossover. Life expectancy at age one exceeds that at age zero for both men and women in India except for Kerala (the only state to experience this crossover in 2000 for men and 1999 for women).

**Conclusions:**

For India, using life expectancy at age zero and under-five mortality rate together may be more meaningful to measure overall health of its people until the crossover. Delayed crossover for women, despite higher life expectancy at birth than for men reiterates that Indian women are still disadvantaged and hence use of life expectancies at ages zero, one and five become important for India. Greater programmatic efforts to control leading causes of death during the first month and 1–59 months in high child mortality areas can help India to attain this crossover early.

## Introduction

Understanding the dynamics of human life and identification of vital processes that help prolong human longevity (often connoted as life expectancy at age zero/birth, $e_{0}^{0})$ has increasingly been gaining importance among researchers and policy makers [1–3]. For measuring overall health of a population, beside routine indicators such as mortality rates at ages below one and age five, life expectancy at age zero is gaining importance as a popular indicator for the same and is also one of the components of the Human Development Index (HDI). During the first half of the twentieth century, life expectancy at age zero in India increased from a low level of less than 25 years to just over 40 years for both sexes. In 2012, estimated life expectancy at age zero for Indian men was 65 years and for Indian women was 68 years [4, 5]. During the early stage of mortality transition, globally, decline in mortality during infancy and at ages 1 to 4 years has contributed more to the initial gains in life expectancy at age zero [6–9]. Until 1980, compared to men, women in India were disadvantaged with regard to life expectancy at age zero [10, 11]. However, for the first time, during 1981–85 life expectancy at age zero for Indian women exceeded that of men [12]. Such findings may sometime be misleading. As a matter of fact, Indian women below age 20 continue to be disadvantaged as a result of major socio-economic discrimination operating against them [13–15]. Thus there is a need to explore alternative indicators of mortality in addition to commonly used indicators such as life expectancy at age zero, mortality during infancy and early childhood to better understand the overall well-being of the population.

Ideally, life expectancy curve should monotonically decline with maxima at age zero. However, in the past, life expectancies at ages one and five have exceeded life expectancy at age zero in India [16]. Higher mortality rates at early ages, especially during infancy and at ages 1 to 4 years might lead to such anomaly. Thus, life expectancy at age zero may fail to provide a true snapshot of the health conditions of the population [17, 18], especially when trying to examine sex differentials in mortality. Therefore, to measure population health, life expectancy at age zero may be appended with the information on mortality during infancy and at ages 1–4 years and/or with life expectancies at higher ages [17, 19]. When life expectancy at age zero falls short of life expectancy at age one, this scenario is referred as ‘imbalance’ in the life table [20]. Highlighting these imbalances may help us recognizing the need for immediate intervention to reduce mortality during infancy and early childhood which in turn would enable the crossover between life expectancies at ages zero, one and five. Besides policy makers, life expectancy at age one is also used to construct Physical Quality of Life Index (PQLI) to measure quality of life of a population. Recently Canudas-Romo and Becker [20] examined crossover for 38 countries, mainly from high income regions. However, they did not include India in their analysis. They show that most of the countries included in the analysis had experienced crossover at a relatively higher levels of life expectancy at age zero (above 73.0 years) [20] which raises an important question, that is, whether achieving high value of life expectancy at age zero is a prerequisite for experiencing the crossover.

The past research [21, 22] has debated that crossover is an artefact of quality of age reporting or any such correlates operating differently on the population sub-groups. However, old age and early life mortality crossover across and within population augur different demographic phenomenon.

In the light of above background, this paper examines levels, patterns, and conditions for crossover between life expectancies at ages zero, one and five for India and selected states representing 90% of the country population in 2011 [23]. The paper also derives a mathematical condition to estimate threshold mortality levels of infant and under-five mortality required for the crossover. The paper specifically quantifies share of infant and under-five mortality in convergence towards crossover between life expectancies at ages zero and one and life expectancies at ages zero and five respectively in India and selected states.

## Materials and Methods

### Ethics Statements

This study is based on publically available secondary data, borrowed from the annual report of Sample Registration System (SRS) Statistical Report, published by Office of the Registrar General of India and Census Commissioner, Ministry of Home Affairs, Government of India, New Delhi. Therefore, no ethical concerns involved in this paper and no ethics review is required for this work.

Initiated during the late 1960s, the Sample Registration System (SRS) has been generating reasonably reliable data on demographic indicators annually for India at the state level [24]. We have used state level SRS age specific death rates (ASDRs) by sex for the period 1979 to 2011 to construct life tables for Indian men and women [16]. Methodology used in this paper to estimate threshold level of infant and under-five mortality required for crossover, constructing life tables and decomposing the change in the gap between life expectancies at ages zero and one and life expectancies at age zero and five is discussed below. Since we have used five years moving average of ASDRs, the analysis is presented for the period 1981 to 2009.

### Threshold level of infant and under-five mortality rates required for crossover

We have used life table functions to derive mathematical conditions (S1 Appendix) to predict a value at which life expectancy at age zero exceeds life expectancy at age one and derived following equations:

$${}_{1}q_{0}\leq \frac{1}{\left(e_{1}^{0}+1-{}_{1}a_{0}\right)}$$ (1)

Where $e_{1}^{0}$ is the life expectancy at age one year and _1_ *a*_0_ is the average number of years lived by those who died before attaining age one. We have obtained life tables using standard software MORTPAK [25] which auto generate values for all columns of life table including _*n*_ *a*_*x*_ subsequently used in the analysis. The MORTPAK selects a suitable model life table based on given age-sex specific death rate (the only input required) and borrows l_x_ (number of person alive at age x) values from the selected model life table. Using these l_x_ values, _*n*_ *a*_*x*_ for each age group are auto generated by the software.

Hence, if infant mortality rate (IMR) is less than or equal to $\frac{1}{\left(e_{1}^{0}+1-{}_{1}a_{0}\right)}$ then life expectancy at age zero must exceed or equalize life expectancy at age one.

Similarly, following equation is derived to predict a value at which life expectancy at age zero exceeds life expectancy at age five:

$${}_{5}q_{0}\leq \frac{5}{\left(e_{5}^{0}+5-{}_{5}a_{0}\right)}$$ (2)

Where $e_{5}^{0}$ is the life expectancy at age five and _5_ *a*_0_ is the average number of years lived by the new-borns who died before attaining age five. _5_ *a*_0_ is the sum of _1_ *a*_0_ and _4_ *a*_1_ as we have assumed that deaths are uniformly distributed within the age group.

Hence, if under-five mortality rate (U5MR) is less than or equal to $\frac{5}{\left(e_{5}^{0}+5-{}_{5}a_{0}\right)}$ then the life expectancy at zero must exceed or equalize life expectancy at age five.

The quantity on the right hand side (RHS) of the Eqs 1 and 2 is defined as the ‘threshold level’ for infant mortality and under-five mortality, respectively. A population must attain IMR equal to or lower than the threshold IMR in order to experience crossover between life expectancies at ages zero and one. Similarly, a population must attain U5MR equal to or lower than the threshold U5MR in order to experience crossover between life expectancies at ages zero and five.

### Constructing life tables for India and states

A preliminary analysis was performed using the published abridged life tables for India [12]. We noted discrepancies such as non-matching of the values on left hand side (LHS) and right hand side (RHS) of the equations (Eqs 3 and 4) in our preliminary analysis on decomposition even though they were mathematical identities with no assumptions involved. We therefore decided to evaluate the published SRS life tables and the methodology used [24, 26] and constructed new set of life tables using five year moving average of SRS age specific death rates [16] later converted into probability of death using Greville’s method. These ASDRs were finally used as input in MORTPAK4.3 [25] to construct life tables.

### Decomposing gaps between $e_{0}^{0}\;:\;e_{1}^{0}$ and $e_{0}^{0}\;:\;e_{5}^{0}$ over time

A decomposition equation is derived after differentiating change in the gap between life expectancies at ages zero and one over time [20, 27]. Using following decomposition equation, gap in life expectancies at ages zero and one is decomposed into two parts- change ‘below age one’ and change ‘above age one’ (S2 Appendix).

$$\begin{array}{l} {}[\{\;{\text{e}}_{0}^{0}\left(t_{2}\right)\;-{\text{e}}_{1}^{0}\left(t_{2}\right)\}\;-\;\{\;{\text{e}}_{0}^{0}\left(t_{1}\right)\;-{\text{e}}_{1}^{0}\left(t_{1}\right)\}]=\left[{}_{1}L_{0}\left(t_{2}\right)\;{-}_{1}L_{0}\left(t_{1}\right)\right]+[{}_{1}d_{0}\left(t_{1}\right)\;{-}_{1}d_{0}\left(t_{2}\right)][\frac{{\text{e}}_{1}^{0}({\text{t}}_{1})+({\text{e}}_{1}^{0}{\text{t}}_{2})}{2}]+\\ \left[\;e_{1}^{0}(t_{1})-e_{1}^{0}(t_{2})\right]\left[\frac{{}_{1}d_{0}\left(t_{1}\right){+}_{1}{\text{d}}_{0}({\text{t}}_{2})}{2}\right] \end{array}$$ (3)

The first two components on the RHS of the Eq 3 represent changes below age one and the third component represents the changes above age one. Below age one component reflects the contribution of mortality condition at ages less than one year and above age one component reflects the contribution of mortality condition at age one and above in realizing the crossover.

We have expanded Eq 3 similarly into more components to decompose change in gap between life expectancies at ages zero and five and obtained following equation (S2 Appendix):

$$\begin{array}{l} {}[\{\;{\text{e}}_{0}^{0}\left(t_{2}\right)\;-{\text{e}}_{5}^{0}\left(t_{2}\right)\}\;-\{{\text{e}}_{0}^{0}\left(t_{1}\right)\;-{\text{e}}_{5}^{0}\left(t_{1}\right)\}]\;=\;\left[{}_{5}\;L_{0}\left(t_{2}\right)\;{-}_{5}L_{0}\left(t_{1}\right)\right]+[{}_{5}\;d_{0}\left(t_{1}\right)\;{-}_{5}d_{0}\left(t_{2}\right)]\left[\frac{e_{5}^{0}(t_{1})+e_{5}^{0}(t_{2})}{2}\right]+\\ \left[e_{5}^{0}(t_{1})-e_{5}^{0}(t_{2})\right]\left[\frac{{}_{5}d_{0}\left(t_{1}\right){+}_{5}d_{0}({\text{t}}_{2})}{2}\right] \end{array}$$ (4)

The first and third components on the RHS of the Eq 4 represent change below age five years and the second component represent change above age five. Below age five components reflects the contribution of mortality condition at ages less than five year and above age five component reflects the contribution of mortality condition at age five and above.

The present analysis is restricted to selected sixteen states of India due to following reasons:

1. In India, the states of Chhattisgarh, Delhi and Jharkhand were formed in 2000 and thus the SRS data is available for less than nine years. As a result, these states were excluded from the analysis.
2. For the state of Jammu and Kashmir data was missing for a few years in between the time period included in the present analysis and hence we excluded this states.

Further, the data on ASDR was missing for few age-groups during the period 1979–81 and for all ages for the year 1990 for the state of Himachal Pradesh. Thus, three years moving averages of ASDR were taken for Himachal Pradesh. We would further like to note that the results for the states of Bihar, Madhya Pradesh and Uttar Pradesh may be less stable as the data for these states until 1999 refers to undivided states and that post 1999 refers to divided states. Furthermore, SRS data for Bihar and Uttar Pradesh is available only after 1980 and as a result estimates for these states were calculated for the period of 1983–09 [16].

## Results

### Crossover between life expectancies at ages ‘zero’ and ‘five’

**Fig 1:**
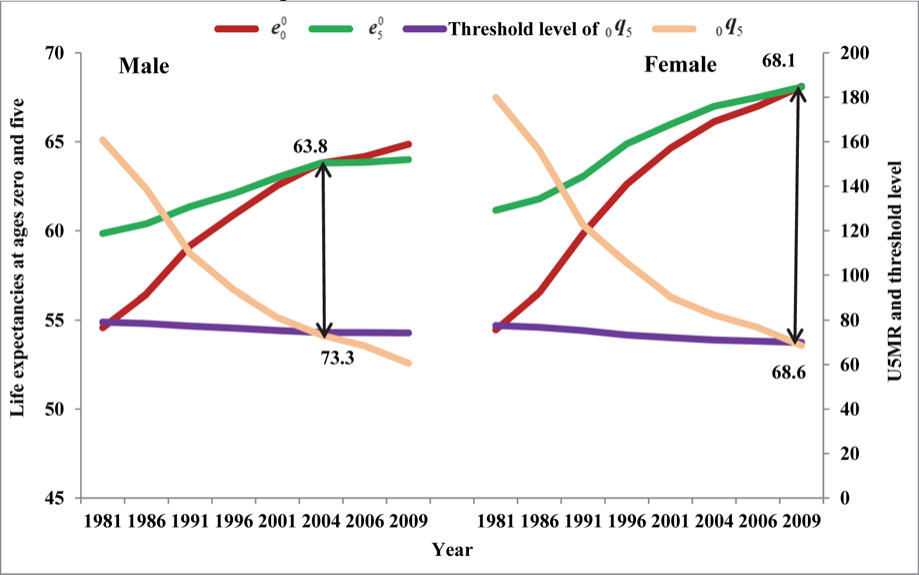
Life expectancies at ages zero and five, under-five mortality rate (U5MR) and threshold level of U5MR required for crossover, India, 1981–2009.

In India, life expectancy at age zero was lower than the life expectancy at age 11.3 years for men and 13.2 years for women during 1981 (S1 Fig). At the national level, India has already experienced crossover between life expectancies at ages zero and five for men in 2004 and for women in 2009 (Fig 1 and S2 Fig). The life expectancy at age zero at the time of crossover was 63.8 years for men and 68.1 years for women (Fig 1). By 2009, eleven Indian states have experienced this crossover for men and nine for women. It needs to be emphasized that compared to Indian women, men experienced the crossover five years earlier (year of crossover, 2009 versus 2004) (Table 1).

**Table 1:** Year of crossover, levels of life expectancies at ages zero and five, under-five mortality rate and its threshold level by sex, India and states.

Male							Female						
India/States	Year	$e_{0}^{0}$	$e_{5}^{0}$	U5MR^2^	Threshold level^1^ ^,^ ^2^	$e_{0}^{0}-e_{5}^{0}$	India/States	Year	$e_{0}^{0}$	$e_{5}^{0}$	U5MR^2^	Threshold^1^ ^,^ ^2^ level	$e_{0}^{0}-e_{5}^{0}$
**Experienced crossover** ^**3**^													
**India**	**2004**	**63.8**	**63.8**	**73.3**	**74.5**	**0.0**	**India**	**2009**	**68.1**	**68.1**	**68.6**	**70.0**	**0.0**
Tamil Nadu	1991	61.3	61.2	74.7	77.6	0.1	Tamil Nadu	1993	64.5	64.5	72.8	73.7	0.0
Andhra Pradesh	2001	62.0	61.8	72.3	76.7	0.2	West Bengal	1997	66.0	65.7	68.1	72.4	0.3
Himachal Pradesh	1994	61.9	61.9	74.8	76.7	0.0	Maharashtra	1994	66.1	66.0	70.4	72.1	0.1
West Bengal	1996	62.3	62.3	75.0	76.2	0.0	Andhra Pradesh	2003	67.5	67.5	69.4	70.6	0.0
Karnataka	1996	62.6	62.4	72.4	76.1	0.2	Karnataka	2001	68.5	68.2	64.9	69.9	0.3
Maharashtra	1991	63.1	63.1	73.0	75.3	0.0	Himachal Pradesh	1999	69.2	69.1	65.8	69.1	0.2
Gujarat	2002	63.9	63.7	71.3	74.6	0.2	Gujarat	2007	69.0	69.0	67.9	69.1	0.0
Haryana	2001	64.8	64.6	69.0	73.7	0.2	Haryana	2009	70.1	70.0	65.0	68.2	0.1
Bihar	2008	65.0	64.8	69.6	73.4	0.2	Punjab	2005	71.0	70.8	62.8	67.5	0.2
Punjab	1991	65.2	65.2	71.0	73.0	0.0							
Rajasthan	2008	65.3	65.3	71.3	72.9	0.0							
**Yet to experience crossover** ^**4**^													
Assam^5^	2009	61.0	61.0	77.9	77.7	-0.1	Bihar	2009	66.0	66.4	76.6	71.7	-0.4
Uttar Pradesh	2009	62.2	62.4	77.6	76.1	-0.2	Orissa	2009	64.4	65.3	84.8	72.8	-0.9
Orissa	2009	62.9	63.5	81.7	74.9	-0.5	Assam	2009	63.4	64.3	86.9	73.9	-0.9
Madhya Pradesh	2009	61.7	62.2	83.2	76.3	-0.5	Rajasthan	2009	69.3	70.4	82.7	67.8	-1.2
							Madhya Pradesh	2009	64.8	66.2	90.9	71.9	-1.4
							Uttar Pradesh	2009	64.5	66.1	93.9	72.0	-1.6

^1^Threshold level of under-five mortality is $5/\left(e_{5}^{0}+5{-}_{5}a_{0}\right)$

^2^Reported in ‘per thousand’

^3^Arranged in descending order of threshold level

^4^Arranged in descending order of $\left(e_{0}^{0}-e_{5}^{0}\right)$

^5^Difference (0.08) is negligible between life expectancies at ages zero and five.

Note: Kerala has achieved the crossover before 1981.

**Fig 2:**
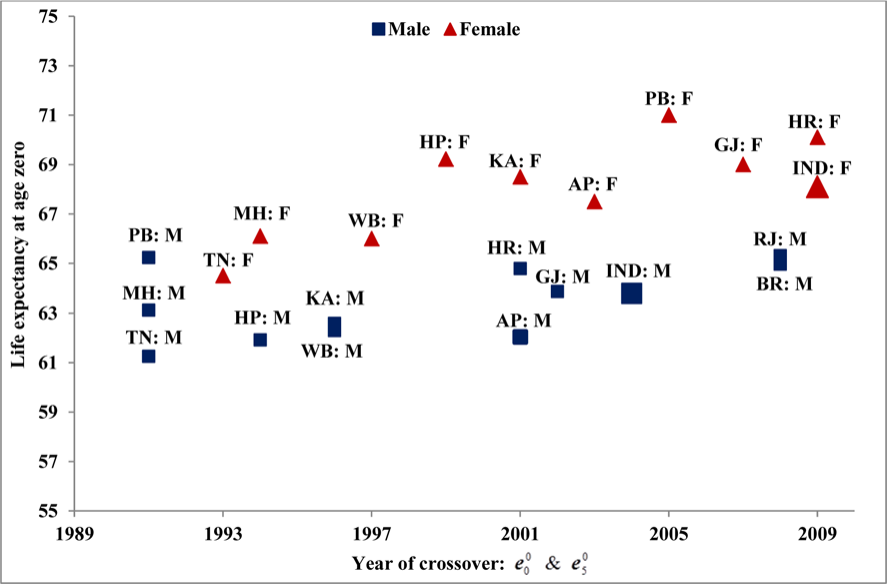
Levels of life expectancy at age zero at the time of crossover between life expectancies at ages zero and five, India and state, 1981–2009.

At the time of crossover between life expectancies at ages zero and five, the year and levels of life expectancy at age zero varied significantly across states for both men and women (Fig 2). While Maharashtra, Punjab, and Tamil Nadu were the first three states to experience this crossover in 1991 for men, Rajasthan and Bihar are the ones who recently in 2008 joined the group. In case of women, Tamil Nadu and Maharashtra were the first to have experienced this crossover during the early 1990s with Haryana being the most recent entry to the group in 2009. At the time of crossover, levels of life expectancy at age zero and the U5MR varied significantly across states for both men and women; For example, for men life expectancy at age zero was 61.3 years in Tamil Nadu and 65.3 years in Rajasthan and the U5MR ranged between 69.0 years in Haryana to 75.0 years in West Bengal. Similarly for women, life expectancy at age zero varied from a low of 64.5 years in Tamil Nadu to 71.0 years in Punjab and the U5MR varied between 62.8 years in Punjab to 72.8 years in Tamil Nadu (Table 1). The results noted for various states confirmed findings for India as a nation, that is, Indian men experiencing crossover earlier than Indian women.

Table 2 shows levels of life expectancies at ages zero and five, U5MR and the estimated threshold levels for the year 2009 for India and states. The observed levels of U5MR for male children in 2009 (the most recent time period for which analysis is done) continues to be higher than the estimated threshold level in four states, viz. Madhya Pradesh, Orissa, Assam, Uttar Pradesh. In addition to these four states, the U5MR in 2009 was higher than the estimated threshold level in Bihar and Rajasthan for female children.

**Table 2:** Life expectancy at age zero and five, under-five mortality rate and its threshold level by sex, India and states, 2009.

Male					Female				
India/States	$e_{0}^{0}$	$e_{5}^{0}$	U5MR	Threshold level	India/States	$e_{0}^{0}$	$e_{5}^{0}$	U5MR	Threshold level
**India**	**64.9**	**64**	**60.6**	**74.3**	**India**	**68.1**	**68.1**	**68.6**	**70**
Madhya Pradesh	61.7	62.2	83.2	76.3	Uttar Pradesh	64.5	66.1	93.9	72
Orissa	62.9	63.5	81.7	74.9	Madhya Pradesh	64.8	66.2	90.9	71.9
Assam	61	61	77.9	77.7	Assam	63.4	64.3	86.9	73.9
Uttar Pradesh	62.2	62.4	77.6	76.1	Orissa	64.4	65.3	84.8	72.8
Rajasthan	65.4	65.1	67.7	73	Rajasthan	69.3	70.4	82.7	67.8
Bihar	65.5	65.1	66	73.1	Bihar	66	66.4	76.6	71.7
Haryana	65.1	64	57.6	74.3	Haryana	70.1	69.9	65	68.2
Gujarat	66	64.9	56.2	73.3	Gujarat	69.9	69.5	62.8	68.6
Andhra Pradesh	63.9	62.4	52.5	76	Andhra Pradesh	68.4	67.4	56	70.6
Karnataka	65.2	63.4	48.3	74.9	Himachal Pradesh	72.1	71.1	52.3	67.1
Himachal Pradesh	67.5	65.7	45.1	72.4	Punjab	72	70.8	50	67.4
Punjab	67.6	65.6	42.2	72.6	Karnataka	70	68.6	48.9	69.5
West Bengal	67.7	65.5	39.8	72.7	West Bengal	71	69	41	69.1
Maharashtra	68.1	65.6	35.1	72.6	Maharashtra	72.1	69.9	37.9	68.2
Tamil Nadu	67.6	64.8	32	73.4	Tamil Nadu	71.4	68.8	33.2	69.2
Kerala	71.4	67.4	13	70.8	Kerala	77.2	73.4	15.4	65.1

Note: Arranged in descending order of U5MR

### Crossover between life expectancies at ages zero and one year

**Fig 3:**
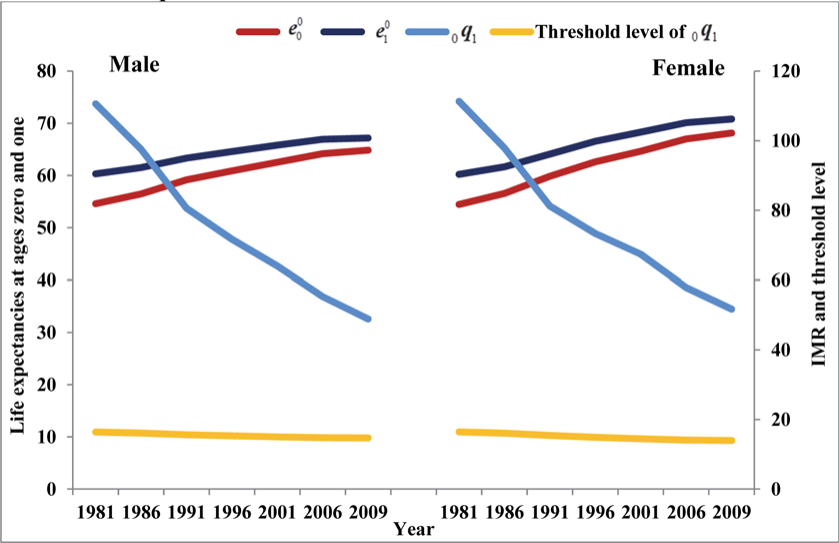
Life expectancies at ages zero and one, infant mortality rate (IMR) and threshold level of IMR required for crossover, India, 1981–2009.

The analysis indicates that India as a nation has yet to experience crossover between life expectancies at ages zero and one (Fig 3). However, Kerala is the only state in India that has already experienced this crossover in the year 2000 for men and in 1999 for women (Table 3 and S3 Fig). At the time of crossover, the life expectancy at age zero was 68.9 years for men and 75.8 years for women in Kerala. Table 3 gives gaps in life expectancies at ages zero and one along with levels of infant mortality and estimated threshold levels for each of the state and India. In the year 2009, difference between life expectancies at ages zero and one was lower in Tamil Nadu (0.9 years for men and 1.1 years for women) and higher in Madhya Pradesh (3.3 years for men and 3.7 years for women).

**Table 3:** Year of crossover, levels of life expectancies at ages zero and one, infant mortality rate and its threshold level by sex, India and states.

Male							Female						
India/States	Year	$e_{0}^{0}$	$e_{1}^{0}$	IMR^2^	Threshold level^1^ ^,^ ^2^	$e_{0}^{0}-e_{1}^{0}$	India/States	Year	$e_{0}^{0}$	$e_{1}^{0}$	IMR^2^	Threshold level^1^ ^,^ ^2^	$e_{0}^{0}-e_{1}^{0}$
**Experienced crossover**													
Kerala	2000	68.9	68.9	14.0	14.3	0.0	Kerala	1999	75.8	75.8	12.6	13.0	0.0
**Yet to experience crossover** ^**3**^													
**India**	**2009**	**64.9**	**67.2**	**48.9**	**14.7**	**-2.3**	**India**	**2009**	**68.1**	**70.8**	**51.7**	**14.0**	**-2.7**
Tamil Nadu	2009	67.6	68.5	27.1	14.4	-0.9	Tamil Nadu	2009	71.4	72.5	29.1	13.6	-1.1
Maharashtra	2009	68.1	69.2	29.0	14.3	-1.0	Maharashtra	2009	72.1	73.4	31.1	13.5	-1.3
West Bengal	2009	67.7	69.0	32.4	14.3	-1.3	West Bengal	2009	71.0	72.5	34.7	13.6	-1.5
Punjab	2009	67.6	69.1	35.7	14.3	-1.5	Punjab	2009	72.0	74.0	39.0	13.4	-1.9
Karnataka	2009	65.2	66.9	40.5	14.8	-1.7	Karnataka	2009	70.0	72.1	41.9	13.7	-2.1
Himachal Pradesh	2009	67.5	69.4	40.6	14.2	-1.9	Himachal Pradesh	2009	72.1	74.5	45.0	13.3	-2.4
Gujarat	2009	66.0	68.1	45.2	14.5	-2.1	Gujarat	2009	69.9	72.4	48.4	13.7	-2.5
Andhra Pradesh	2009	63.9	66.1	47.4	14.9	-2.2	Andhra Pradesh	2009	68.4	71.0	50.4	13.9	-2.6
Haryana	2009	65.1	67.3	48.1	14.7	-2.3	Bihar	2009	66.0	68.6	52.6	14.4	-2.7
Bihar	2009	65.5	68.0	50.5	14.5	-2.5	Haryana	2009	70.1	73.0	52.7	13.6	-2.9
Assam	2009	61.0	63.8	59.1	15.5	-2.8	Assam	2009	63.4	66.6	62.4	14.9	-3.2
Rajasthan	2009	65.4	68.3	56.7	14.5	-2.9	Rajasthan	2009	69.3	72.7	60.6	13.6	-3.5
Uttar Pradesh	2009	62.2	65.3	61.3	15.1	-3.0	Orissa	2009	64.4	67.9	65.4	14.6	-3.5
Orissa	2009	62.9	66.2	63.6	14.9	-3.3	Uttar Pradesh	2009	64.5	68.0	65.5	14.5	-3.5
Madhya Pradesh	2009	61.7	65.0	65.0	15.2	-3.3	Madhya Pradesh	2009	64.8	68.5	67.5	14.4	-3.7

^1^Threshold level of infant mortality is $1/\left(e_{1}^{0}+1{-}_{1}a_{0}\right)$

^2^Reported in ‘per thousand’

^3^Arranged in descending order of $\left(e_{0}^{0}-e_{1}^{0}\right)$

### Convergence towards crossover between life expectancies at ages zero and one and life expectancies at ages zero and five: Relative share of IMR and U5MR

**Fig 4:**
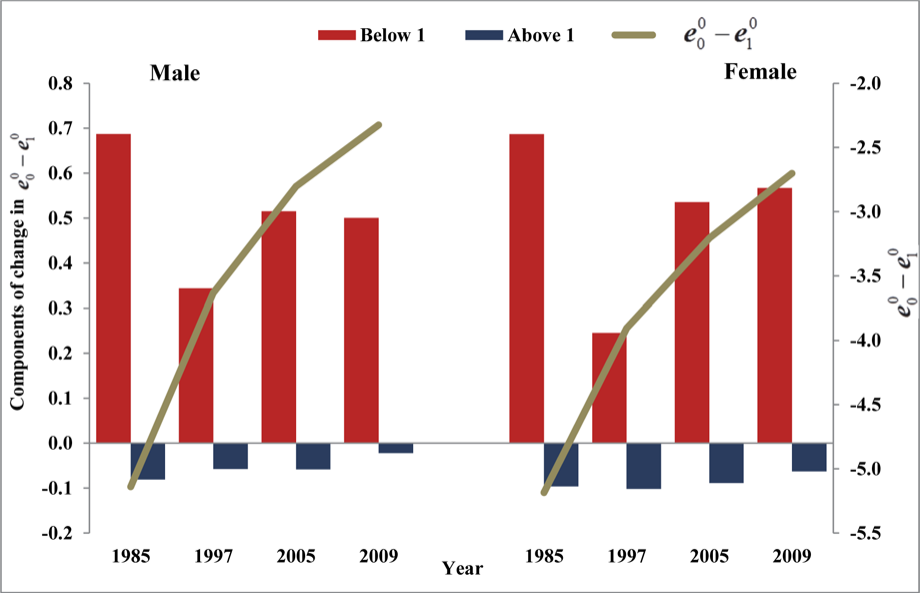
Gaps in life expectancies at ages zero, one and five and the components of change in the gap over time, India, 1981–2009.

**Fig 5:**
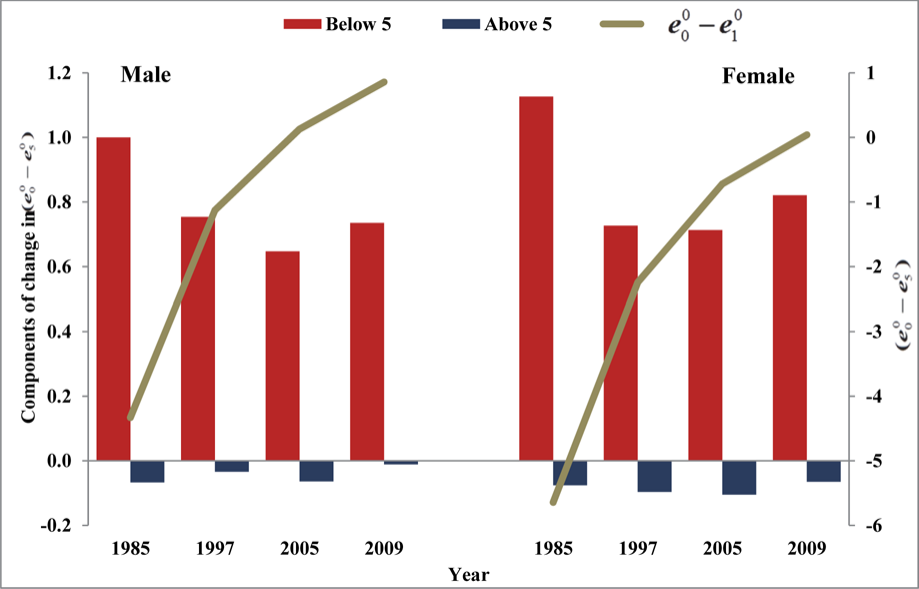
Gap in life expectancies at ages zero and five and the components of the change in the gap over time, India, 1981–2009.

The decomposition of change in $e_{0}^{0}-e_{1}^{0}$ and $e_{0}^{0}-e_{5}^{0}$ allows to examine relative share of each component towards crossover. The two components of the change included in the analysis are: changes ‘below age one’ and ‘above age one’ when analysing crossover at age one. Likewise, two components included in the analysis are ‘below age five’ and ‘above age five’ when examining crossover at age five. These components have been calculated for each five years interval during period 1981 to 2009 for India (Figs 4 and 5) and states (S3 and S4 Tables). Although the results do not reveal any specific pattern as relative share of the components fluctuates over time and across states. Nonetheless, it is noted that compared to mortality at the older ages, mortality during infancy and at ages 1–4 years has relatively dominating effects in reducing the gaps between life expectancies at ages zero, one and five.

Results show that the relative share of reduction in mortality below age one is mostly positive while that of above age one is mostly negative. This means that mortality reduction during the infancy has led to the convergence towards crossover between life expectancies at ages zero and one. At the national level, for men and women, mortality below age one has contributed 113.4% and 116.2%, respectively, towards this convergence during 1981–85. The changes in mortality above age one have actually widened these gaps by nearly 13.4% for men and 16.2% for women during the same time (S3 Table). In the recent period (2005–09), improvements in mortality below age one has contributed 104.6% for men and 112.5% for women towards crossover. On the other hand, improvements in mortality at ages above one have actually widened these gaps by 4.6% for men and 12.5% for women during the same period. The share of improvement in mortality below age one has, however, reduced during 1980–2009. At the same time, improvement in mortality above age one has been gaining increasing importance in the convergence towards crossover, probably due to relative increase in the adult mortality [28, 29].

State level analysis indicates somewhat similar patterns to that observed at the national level (Table 4 and S3 Table). The analysis shows that improvement in mortality below age one has contributed more in the convergence towards crossover between life expectancies at ages zero and one by about 12.9% in Assam to about 142.1% in Haryana for men during 1981–85 (S3 Table). The corresponding range for 2009 was observed at 89.9% in Haryana to 116.6% in Gujarat. This means that the relative contribution of age below one has slowed down across states. The share of age below one varied from 24.5% in Karnataka to 179.0% in Tamil Nadu during the 1981–85 and widened to a low of 82.2% in Himachal Pradesh to 338.3% in Assam. Table 4 summarizes state specific minimum and maximum share of mortality below age one and above age one in convergence towards crossover.

**Table 4:** Lower (L) and higher (H) value of the share of mortality below age one and above age one in convergence towards crossover between life expectancies at ages zero and one, 1981–09.

Year	Male		Female	
	Below 1 L/H (State/State)	Above 1 L/H (State/State)	Below 1 L/H (State/State)	Above 1 L/H (State/State)
**1981–85**	12.9/142.1(AS/HR)	-42.1/87.1(HR/AS)	24.5/179.0(KA/TN)	-79.0/75.5(TN/KA)
**1985–89**	72.8/158.3(KA/WB)	-58.3/27.2(WB/KA)	-552.2/133.6(OR/AP)	-33.6/652.2(AP/OR)
**1989–93**	84.8/220.6(HP/RJ)	-120.6/15.2(RJ/HP)	104.1/466.1(HR/WB)	-366.1/-4.1(WB/HR)
**1993–97**	65.5/2266.7(PB/RJ)	-2166.7/34.5(RJ/PB)	-1142.9/695.2(AS/PB)	-595.2/1242.9(PB/AS)
**1997–01**	64.3/187.8(KA/AP)	-87.8/35.7(AP/KA)	-370.5/677.8(HR/KA)	-577.8/470.5(KA/HR)
**2001–05**	-303.6/125.4(AS/MP)	-25.4/403.6(MP/AS)	74.4/158.5(HP/BR)	-58.5/25.6(BR/HP)
**2005–09**	89.9/116.6(HR/GJ)	-16.6/10.1(GJ/HR)	82.2/124.2(HP/MP)	-283.3/17.8(AS/HP)

Note: Figures are based on state level comparison.

The analysis related to share of improvements in mortality at ages below five and above five for India and states confirmed to the patterns observed for share below age one and above age one, for both India as a nation and for states (Fig 5, Table 5 and S4 Table).

**Table 5:** Lowest (L) and highest (H) value of the share mortality below age five and above age five in convergence towards crossover between life expectancies at ages zero and five, 1981–09.

Year	Male		Female	
	Below 5 L/H (State/State)	Above 5L/H (State/State)	Below 5 L/H (State/State)	Above 5 L/H (State/State)
**1981–85**	11.3/137.8(AS/HR)	-37.8/88.7(HR/AS)	20.4/167.1(KA/TN)	-67.1/79.6(TN/KA)
**1985–89**	87.7/201.5(AP/OR)	-101.5/12.3(OR/AP)	-0.4/134.2(OR/BR)	-34.2/100.4(BR/OR)
**1989–93**	88.5/146.5(HP/AS)	-46.5/11.5(AS/HP)	103.0/152.7(MH/HP)	-52.7/-3.0(HP/MH)
**1993–97**	79.5/159.2(PB/RJ)	-59.2/20.5(RJ/PB)	-248.1/300.9(HR/AP)	-200.9/348.1(AP/HR)
**1997–01**	92.1/138.8(HR/TN)	-38.8/7.9(TN/HR)	-5.7/137.4(PB/AS)	-37.4/105.7(AS/PB)
**2001–05**	-308.1/121.7(AS/MP)	-21.7/408.1(MP/AS)	73.8/154.7(HP/BR)	-54.7/26.2(BR/HP)
**2005–09**	88.3/115.0(HR/OR)	-15.0/11.7(OR/HR)	-66.0/111.9(AS/MP)	-11.9/166.0(MP/AS)

Note: Figures are based on state level comparison.

## Discussion and Conclusions

The Indian life tables reveal that a newly born child has shorter longevity then does a child who has survived to age one and/or five years. The life expectancy at age zero for women is three years greater than that for men in 2009 and at the same time the U5MR for female children was 10% higher than the U5MR for male children during the same year implying that the life expectancy at age zero alone may hide the widespread gender discrimination and disadvantages for female children at early ages of life. Such scenarios make us question about the utility of life expectancy at age zero alone as a summary indicator for measuring the overall health of the population in its true sense.

Our analysis using model life tables developed by Coale and Demeny (CD) [30] and United Nations (UN) [31], show that at the time of crossover between life expectancies at ages zero and five, value of life expectancy at age zero ranged from about 57 years (in UN Far East Asian model life tables) to nearly 69 years (in CD South model and UN South Asian model life table) for males and from about 62 years (in UN Far East Asian model life tables) to 72 nearly years (in CD South model and UN South Asian model life table) for females (Table A in S2 Table).

At the time of crossover between life expectancies at ages zero and one, value of life expectancy at age zero ranged from about 74 years (in UN Far East Asian model life tables) to nearly 84 years (in Coale and Demeny South model life tables) for males and from about 78 years (in Coale and Demeny West model life tables) to nearly 87 years (in Coale and Demeny South model life tables) for females (Table B in S2 Table). Compared to other model life tables, crossover occurred at lower levels of life expectancy at age zero in the UN Far East Asian model life tables. Similar observations were made in a study conducted in Russia [20]. The corresponding mortality levels at crossover indicate that such differences could be the result of higher all-cause mortality and/or cause specific mortality during adulthood and/or some specific relationship between mortality during infancy, early childhood and adulthood [29, 32].

In order to avoid annual fluctuations in the rates, we have reconstructed new set of life tables using five year moving average of ASDRs. These new life tables also overcome the discrepancy found in the decomposition analysis based on SRS abridged life tables.

During 1996–2006, India’s official life tables from SRS not only showed a rather spurious rise in the child mortality but also the ratio of mortality at ages 1–4 years to mortality during infancy did not follow any model life table pattern. Previous research [26] examined this unusual revelation for major states in India and noted that these results were outcome of methodological errors made at the time of constructing the life tables. The authors then reconstructed a new set of life tables using the same methodology and found that the new estimates were comparable with the pattern globally observed for infant and child mortality.

Various researchers [24, 33, 34] in the past have examined quality of SRS estimates with respect to under and/or over reporting of deaths for ages above five only. For the present work, however, we have not made any adjustments in the SRS rates with respect to completeness as this may virtually have no or negligible effect on the resultant age patterns of mortality and thereby the crossover between life expectancies at ages zero, one and five will not be affected. Our work proposes new set of mathematical equations to estimate threshold level of IMR and U5MR required for crossover between life expectancies at ages zero and one; and zero and five respectively. These newly derived equations have been used to estimate the threshold level of IMR and U5MR for India and its selected states to examine the crossover.

India as a nation experienced crossover between life expectancies at ages zero and five in 2004 for men and 2009 for women; of sixteen states included in the analysis, eleven and nine have already had this experience for men and women, respectively. Further, in all instances men experienced this crossover on an average four years earlier than did Indian women. Not only this, Indian men experienced this crossover at relatively lower levels of life expectancy at age zero (61–65 years) compared to Indian women (65–70 years). To best of our knowledge no other country has demonstrated a crossover between life expectancies at ages zero and five. However, our analysis based on model life tables indicates that the level of life expectancy at age zero ranges between 57–60 years for men and 62–72 years for women at the time of crossover.

In spite of its best efforts to meet the Millennium Development Goals, especially in the last 10–15 years, the life expectancy at age one continues to exceed that at age zero for India as a nation (meaning that country has yet to experience crossover between life expectancies at ages zero and one). Kerala is the only state which has experienced this crossover in the beginning of the present century; 2000 for men and 1999 for women. Thus, there is a need to further intensify the efforts to reduce IMR to its threshold level to enable India and the remaining states to experience the crossover between life expectancies age zero and one.

Not only majority of the infant deaths are concentrated during neonatal period but also the leading causes of deaths among neonates are quite different than those for death at ages 1–59 months [35, 36]. Further, a recent study [15] shows that in India, about 251 (42%) districts lag behind the relevant goal for neonatal mortality in 2012 which may largely be responsible for India’s inability to experience the crossover at ages zero and one. Therefore, a separate analysis investigating the role of neonatal mortality in the crossover between life expectancies at ages zero, one and five for India would certainly be more insightful.

The states in India are at different stages as the expected threshold value of IMR required for this crossover varies across states and as a result a few of the Indian states may experience this crossover before 2020 (linearly extrapolated) while others may have to wait longer. Thus it appears that the programmatic efforts in India over the past decades have led to relatively faster improvement in mortality during infancy and early childhood among male children compared to the female children [15]. In a population like India where gender discrimination at early ages in life are extensively and commonly prevalent, there is a need to consider other indicators of mortality in addition to life expectancy at age zero. As paper demonstrate, despite higher life expectancy at age zero for female, crossover for women got delayed by almost 4 to 5 years reinstating the fact that the women in India are grossly disadvantage during the early ages of lives. In this context, relative values of life expectancies at age zero, one and five become important. Exploring crossover over time would help us indicate that when life expectancy become highest at age zero and hence the life expectancy at age zero becomes a single health indicator of the population.

The analysis indicates that improvements in mortality ‘below age one’ and ‘below age five’ have dominating effects in convergence towards crossover between life expectancies at ages zero, one and five for India and its states. These findings are encouraging in the context of recent efforts made by the government in the form of implementation of Child Survival and Safe Motherhood programme (CSSM), National Rural Health Mission (NRHM), Janani Suraksha Yojana (JSY) etc. aiming to reduce persistent higher infant and under-five mortality resulting in improved child survival. Although, many districts in India have made remarkable progress in this direction, 222 Indian district lag considerably behind in achieving the Millennium Development Goals [15]. At the same time female children in India continue to be greatly disadvantaged than the male children with respect to under-5 mortality and this phenomenon is seen in almost all parts of the country. This highlights for concentrated efforts in the lagging areas to expedite improvement in child survival enabling crossover.

Of nearly eight million child deaths among children under age five in 2010 globally, 64% were attributable to infectious diseases (Pneumonia, Measles and Diarrhoea). India was one of the five countries accounting for over half of under-five deaths in 2010 [37]. The recently published RGI report on causes of deaths (2004–06) in India [38] and past studies [39] revealed that Diarrhoeal diseases, Pneumonia and Injuries together accounted for over half of the deaths among children at ages 1–4 years in India. Further, Prematurity and low birth weight, Pneumonia, Birth asphyxia & birth trauma, Diarrhoeal diseases and Other non-communicable diseases are the top five causes of deaths together accounting for 73.1% of total deaths during infancy. Among neonates, Prematurity and low birth weight, Neonatal infections and Birth asphyxia & birth trauma are the top causes of deaths and accounted for 78% of all neonatal deaths in India in 2005[39]. Additionally, reduction in poverty and improvements in education, hygiene and sanitation access have found to be strongly associated with the improvements in survival, especially at younger ages [40–42]. Thus, focus on strategies towards these leading causes of deaths and directing resources towards improvements in access to basic amenities including access to safe drinking water and sanitation services might expedite improvements in survival, especially among young children. Over 300 Indian districts, spread across all states, have significantly higher mortality for female children under age five than the male children [15] indicating for need to intensify the efforts to reduce this gap. If implemented effectively, these would enhance India’s chances of accelerating the journey of crossover between life expectancies at ages zero, one and five, especially in areas that have yet to experience crossover.

## Supporting Information

S1 Appendix Condition for the crossover in life expectancies: Estimating threshold levels of infant and under-five mortality. (PDF)

S2 AppendixDecomposition of the change in life expectancies over time: $e_{0}^{0}\;/\;e_{1}^{0}$ and $e_{0}^{0}\;/\;e_{5}^{0}$.(PDF)

S1 Fig Life expectancies by age and sex, 1981 and 2009, India. (TIF)

S2 Fig Life expectancies at ages zero, one and five and year of crossover, India, 1981–2009. (TIF)

S3 Fig Life expectancies at ages zero, one and five and year of crossover, Kerala, 1981–2009. (TIF)

S1 Table Life expectancies at ages zero, one and five, India and states, 1981 and 2009. (PDF)

S2 Table A: Levels of crossover between life expectancies at ages zero and five and corresponding (_1_ *q*_0_), under-five (_0_ *q*_5_) and adult mortality (_45_ *q*_15_), various model life tables. B: Levels of crossover between life expectancies at ages zero and one and corresponding infant (_1_ *q*_0_), under-five (_0_ *q*_5_) and adult mortality (_45_ *q*_15_), various model life tables. (PDF)

S3 Table Components (in %) of change in gap between life expectancies at ages zero and one, India and States, 1981–09. Arranged in descending order of “Below age one” component in the period 2005–09.
(PDF)

S4 Table Components (in %) of change in gap between life expectancies at ages zero and five, India and States, 1981–09. Arranged in descending order of “Below age five” component in the period 2005–09.
(PDF)
