# Supplementary material for: Threshold Levels of Infant and Under-Five Mortality for Crossover between Life Expectancies at Ages Zero, One and Five in India: A Decomposition Analysis
Source: PLoS One. 2015 Dec 18;10(12):e0143764. doi: 10.1371/journal.pone.0143764 (PMC4684288; doi:10.1371/journal.pone.0143764)
Supplement: S1 Appendix — (PDF) [file pone.0143764.s001.pdf]

S1A Appendix: Condition for the crossover in life expectancies: Estimating threshold levels of infant and under-five mortality

The derivation of the mathematical condition, using life table functions, to understand the level of infant and under-five mortality at time of crossover e.g. when $\left(e_0^0 \geq e_1^0\right)$ and $\left(e_0^0 \geq e_5^0\right)$ are given below:

A simple mathematical relation is derived from a basic definition of life expectancy given as:

$$e_x^0(t) \ = \frac{\int_x^w l(a,t)\,da}{l(x,t)}$$

Let $l(0,t) = 1$

$$e_0^0\big(t\big) = \int_0^1 l(x,t)\,dx + \int_1^w l(x,t)\,dx$$

$$e_0^0 = {}_1L_0 + e_1^0\left[1 - {}_1d_0\right] \qquad (1)$$

Now, we have,

$$\begin{aligned} {}_nL_x &= \ n\,l_{x+n} + {}_nA_x \\ &= n\,\mathrm{l}_{x+n} + {}_na_x\,{}_nd_x \end{aligned} \qquad (2)$$

For the age group 0-1, equation (2) can be written as

$${}_1L_0 = l_1 + {}_1a_0\,{}_1d_0 \qquad (3)$$

Now, from equation (1) we get

$$e_0^0 = {}_1L_0 + e_1^0\left[1 - {}_1d_0\right]$$

$$= [l_1 + {}_1a_0 \ {}_1d_0] + e_1^0 - e_1^0 \ {}_1d_0$$ *(Using equation 3)*

$$= l_1 + {}_1a_0 \left(l_0 - l_1\right) + e_1^0 - e_1^0 (l_0 - l_1)$$

$$= l_1 + {}_1a_0 l_0 - {}_1a_0 \, l_1 + e_1^0 - e_1^0 \, l_0 + e_1^0 l_1$$

For simplification let $l_0 = 1$

$$e_0^0 = l_1 + {}_1a_0 - {}_1a_0 \, l_1 + e_1^0 l_1$$
$$= {}_1a_0 + l_1 \ (1 + e_1^0 - {}_1a_0)$$
$$= {}_1a_0 + \left(1 - {}_1q_0\right)(1 + e_1^0 - {}_1a_0) \qquad (l_1 = 1 - {}_1q_0)$$

For crossover,

$$e_0^0 \geq e_1^0$$

$${}_1a_0 + \left(1 - {}_1q_0\right)(1 + e_1^0 - {}_1a_0) \geq \boldsymbol{e}_1^0$$

$${}_1q_0 \leq \frac{1}{\left(e_1^0 + 1 - {}_1a_0\right)}$$

Hence if IMR is less than or equal to $\frac{1}{\left(e_1^0 + 1 - {}_1a_0\right)}$ then only e_0^0 will be greater than equal to e_1^0.

Similarly we can also write equation 1 as follows:

$$e_0^0\left(t\right) = \int_0^5 l\left(x,t\right) dx + \int_5^w l\left(x,t\right) dx$$

$$e_0^0 = {}_5L_0 + e_5^0\left[1 - {}_5d_0\right]$$

$$= \left[5l_5 + {}_5a_0\,{}_5d_0\right] + e_5^0 - e_5^0\,{}_5d_0 \qquad \textit{(Using equation 2)}$$

$$= \ 5l_5 + {}_5a_0(l_0 - l_5) + e_5^0 - e_5^0(l_0 - l_5)$$

$$= 5l_5 + {}_5a_0l_0 - {}_5a_0l_5 + e_5^0 - e_5^0l_0 + e_5^0l_5)$$

For simplification let $l_0 = 1$

$$e_0^0 = 5l_5 + {}_5a_0 - {}_5a_0l_5 + e_5^0l_5)$$

$$= {}_5a_0 + l_5(5 + e_5^0 - {}_5a_0)$$

$$= {}_5a_0 + (1 - {}_5q_0)(5 + e_5^0 - {}_5a_0) \qquad \left(l_5 = \ 1 - {}_5q_0\right)$$

For crossover,

$$e_0^0 \geq e_5^0$$

$${}_5a_0 + \left(1 - {}_5q_0\right)\left(5 + e_5^0 - {}_5a_0\right) \geq e_1^0$$

$${}_5q_0 \leq \frac{5}{\left(e_5^0 + 5 - {}_5a_0\right)}$$

Hence if under-five mortality is less than or equal to $\frac{5}{\left(e_5^0 + 5 - {}_5a_0\right)}$ then only $\boldsymbol{e_0^0}$ will be greater than equal to $\boldsymbol{e_5^0}$.
