# Supplementary material for: Threshold Levels of Infant and Under-Five Mortality for Crossover between Life Expectancies at Ages Zero, One and Five in India: A Decomposition Analysis
Source: PLoS One. 2015 Dec 18;10(12):e0143764. doi: 10.1371/journal.pone.0143764 (PMC4684288; doi:10.1371/journal.pone.0143764)
Supplement: S2 Appendix — (PDF) [file pone.0143764.s002.pdf]

S1B Appendix: Decomposition of the change in life expectancies over time: $\boldsymbol{e_0^0 / e_1^0}$ and $\boldsymbol{e_0^0 / e_5^0}$

The gap in the life expectancy e.g. $e_0^0 - e_1^0$ and $e_0^0 - e_5^0$ are decomposed into their components. To find out the decomposition equation, the change in the gap over time between e_0^0 and e_1^0 were differentiated (first derivative with respect to time). And then the equation is simplified for estimation by using decomposition of Kitagawa [28].

With simple mathematics, we can derive a decomposition equation using life table functions:

$$e_0^0 = {}_1L_0 + e_1^0\left[1 - {}_1d_0\right]$$

$$= e_1^0(t) - e_1^0(t)\,{}_1d_0(t) + {}_1L_0(t)$$

Difference between the life expectancies at ages zero and age one is given by:

$$\Delta e_{0-1}^0(t) = e_0^0(t) - e_1^0(t)$$

$$= e_1^0(t) - e_1^0(t)_1\, d_0(t) + {}_1L_0(t) e_1^0(t)$$

$$= {}_1L_o(t) \; - e_1^0(t)_1\, d_o(t)$$

Now, differentiate the above equation with respect to time, we get

$$d[\Delta e_{0-1}^0(t)]/dt \;=\; d\left[{}_1L_o(t)\right]/dt \; - e_1^0(t)\, d\left[{}_1d_o(t)\right]/dt \; -{}_1d_o(t)\, d[e_1^0(t)]/dt$$

This equation can be more simplified for estimation by using decomposition of Kitagawa as:

$$[\{ e_0^0(t_2) - e_1^0(t_2)\} - \{e_0^0(t_1) - e_1^0(t_1)\}] = [{}_1L_0(t_2) - {}_1L_0(t_1)] + [{}_1d_0(t_1) - {}_1d_0(t_2)]\left[\frac{e_1^0(t_1)+e_1^0(t_2)}{2}\right] +$$

$$\left[e_1^0(t_1) - e_1^0(t_2)\right]\left[\frac{{}_1d_0(t_1) + {}_1d_0(t_2)}{2}\right]$$

here, on right hand side, first two components reflects the changes ‘below age one’ and third component represents the changes ‘above age one’.

Similarly, we have expanded the above equation into more components to decompose the change in the gap between e_0^0 *and* e_5^0.

The difference between the life expectancies at ages zero and five is given by:

$$\Delta e_{0-5}^0(t_1) = \left[{}_5L_0(t_1)\right] - e_5^0(t_1)\,{}_5d_0(t_1)$$

$$\Delta e_{0-5}^0(t_2) = \left[{}_5L_0(t_2)\right] - e_5^0(t_2)\,{}_5d_0(t_2)$$

After differentiating with respect to time and using the Kitagawa [28] decomposition equation, we get

$$[\{ e_0^0(t_2) - e_5^0(t_2)\} - \{e_0^0(t_1) - e_5^0(t_1)\}] = [{}_5L_0(t_2) - {}_5L_0(t_1)] + [{}_5d_0(t_1) - {}_5d_0(t_2)]\left[\frac{e_5^0(t_1)+e_5^0(t_2)}{2}\right] +$$

$$\left[e_5^0(t_1) - e_5^0(t_2)\right]\left[\frac{{}_5d_0(t_1) + {}_5d_0(t_2)}{2}\right]$$

Here, on right hand side, sum of first and third components shows the effect of changes in mortality ‘below age 5’ years and second component term represent the role of mortality changes ‘above age five’.
