# Supplementary figures and images for: Threshold Levels of Infant and Under-Five Mortality for Crossover between Life Expectancies at Ages Zero, One and Five in India: A Decomposition Analysis
Source: PLoS One. 2015 Dec 18;10(12):e0143764. doi: 10.1371/journal.pone.0143764 (PMC4684288; doi:10.1371/journal.pone.0143764)

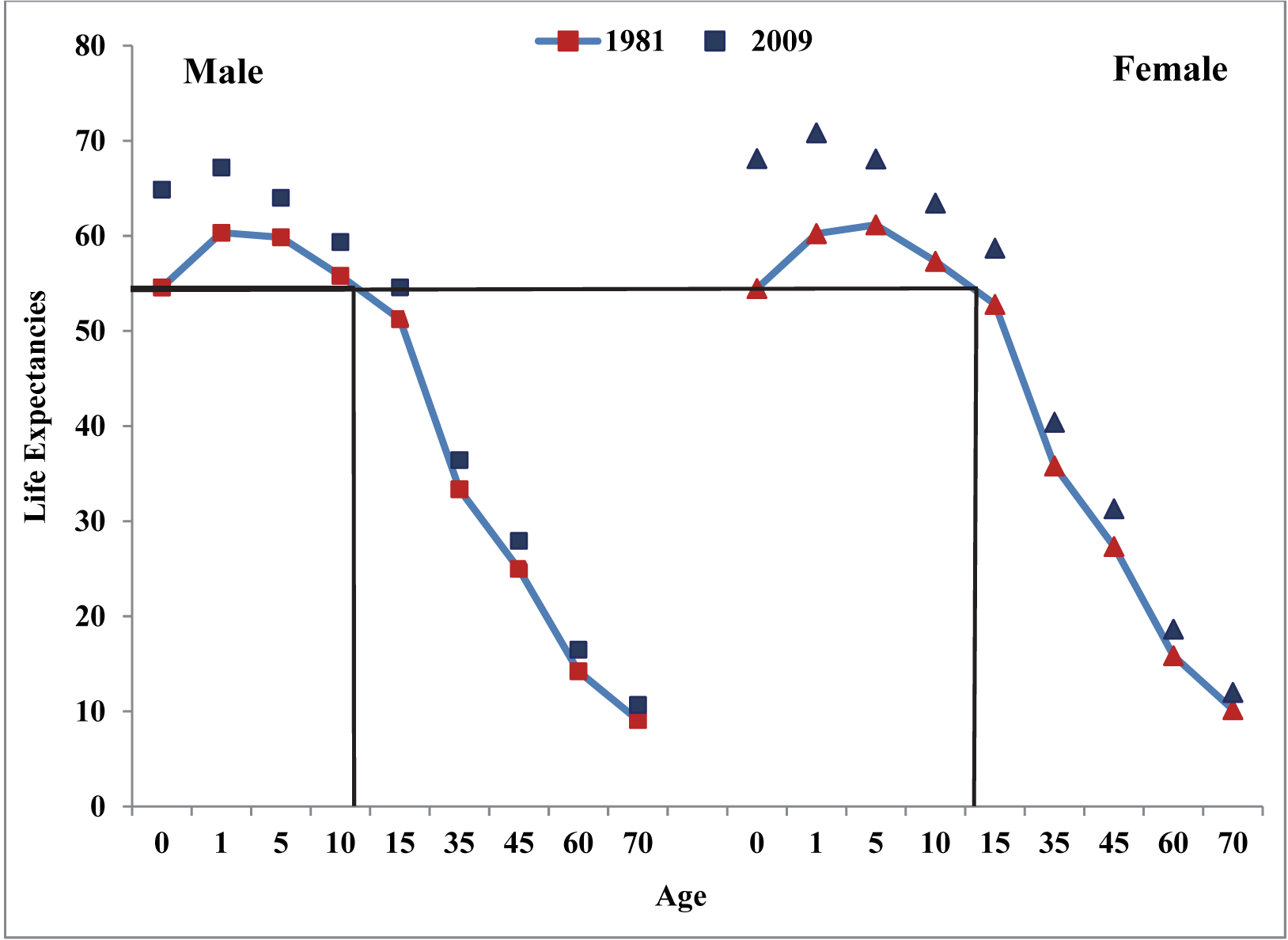

Supplement: S1 Fig — (TIF) [file pone.0143764.s003.tif]

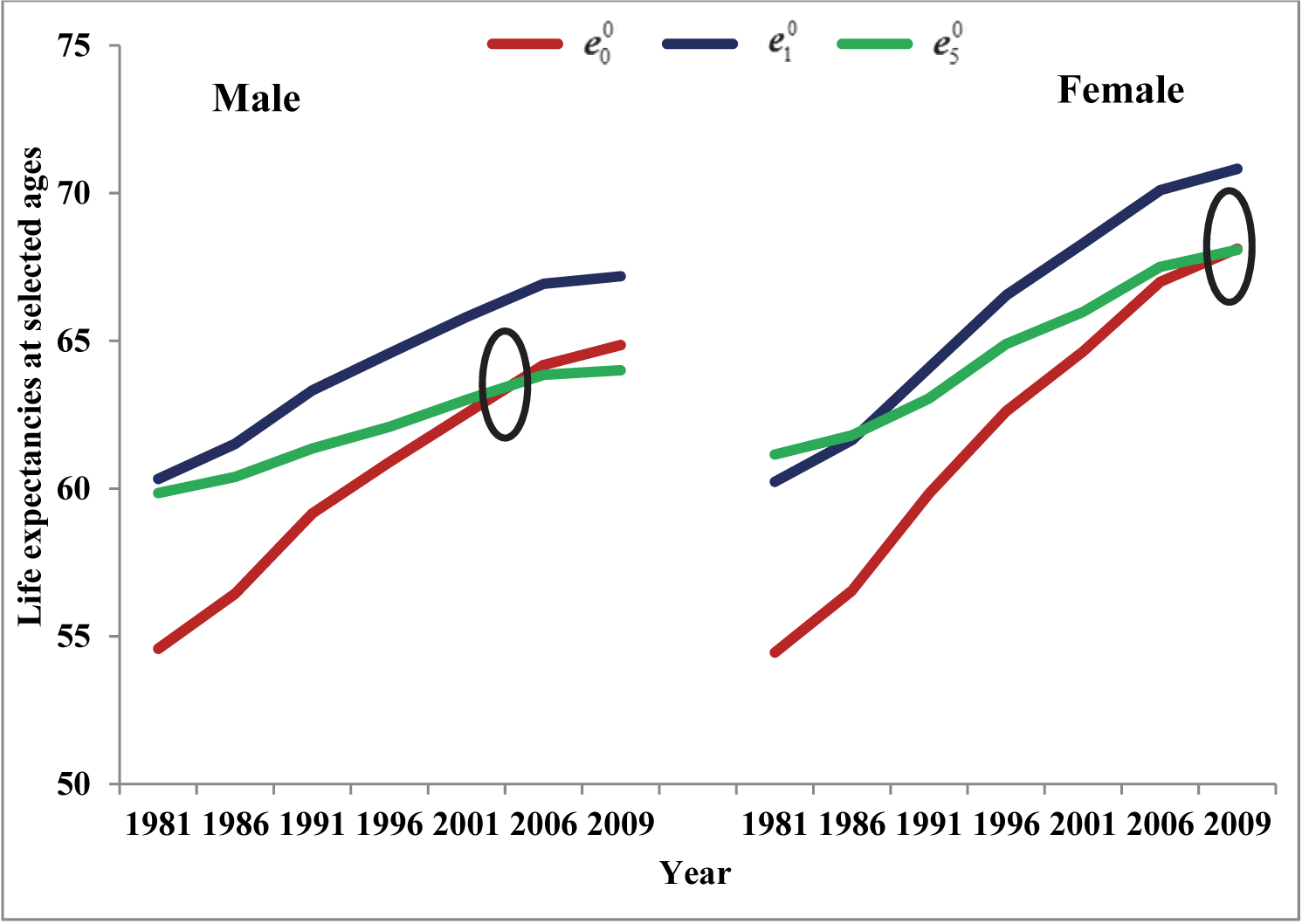

Supplement: S2 Fig — (TIF) [file pone.0143764.s004.tif]

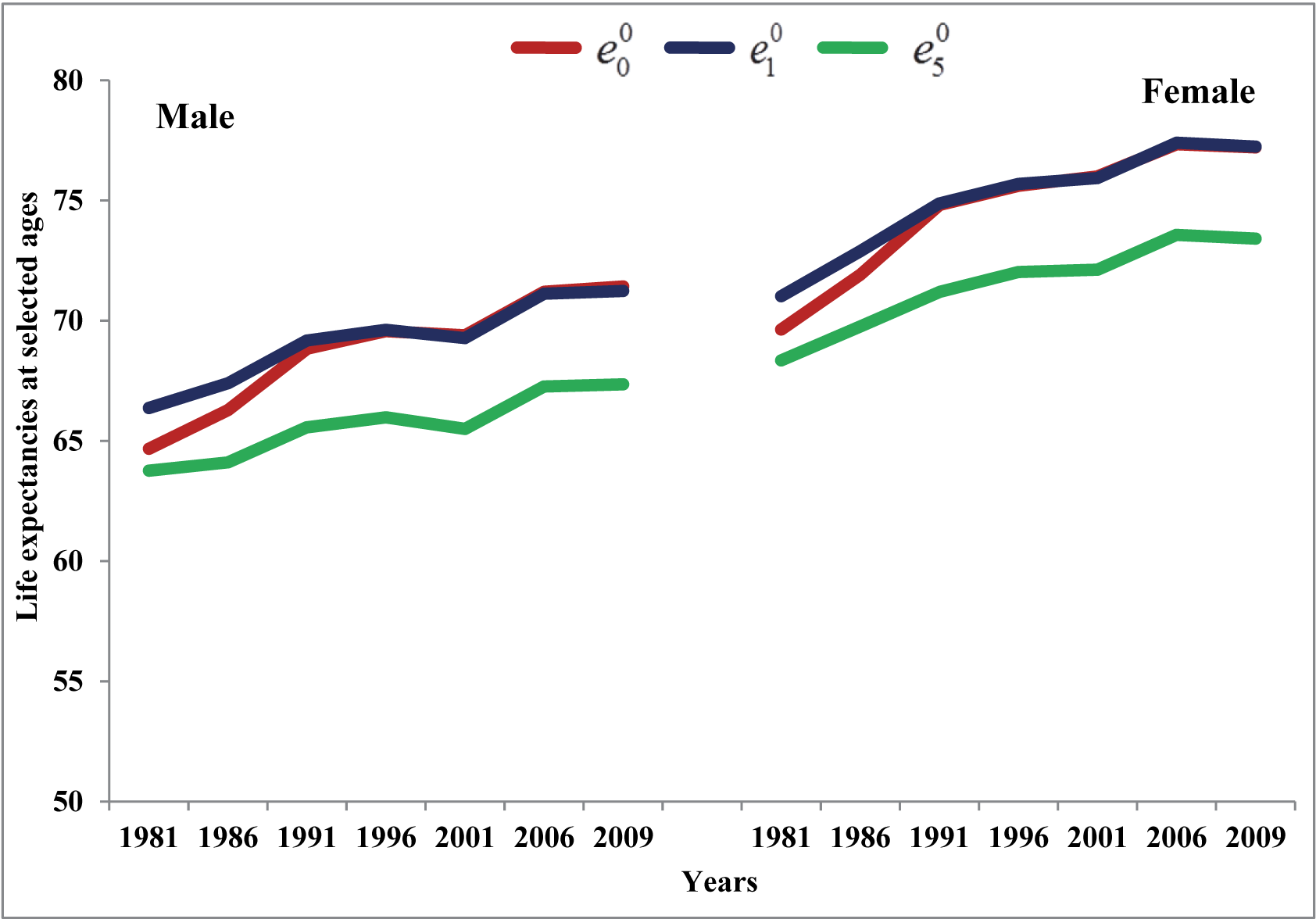

Supplement: S3 Fig — (TIF) [file pone.0143764.s005.tif]
