# Supplementary material for: Threshold Levels of Infant and Under-Five Mortality for Crossover between Life Expectancies at Ages Zero, One and Five in India: A Decomposition Analysis
Source: PLoS One. 2015 Dec 18;10(12):e0143764. doi: 10.1371/journal.pone.0143764 (PMC4684288; doi:10.1371/journal.pone.0143764)
Supplement: S1 Table — (PDF) [file pone.0143764.s006.pdf]

S1 Table: Life expectancies at ages zero, one and five, India and states, 1981 and 2009

India/States	Year	Male			Female		
		e_0^0	e_1^0	e_5^0	e_0^0	e_1^0	e_5^0
**India**	1981	54.6	60.3	59.8	54.5	60.2	61.2
	2009	64.9	67.2	64.0	68.1	70.8	68.1
**Andhra Pradesh**	1981	55.2	59.9	58.5	58.5	62.6	61.5
	2009	63.9	66.1	62.4	68.4	71.0	67.4
**Assam**	1981	52.6	57.8	57.5	52.3	57.0	57.1
	2009	61.0	63.8	61.0	63.4	66.6	64.3
**Bihar**	1983	54.1	59.2	59.5	51.0	56.2	57.6
	2009	65.5	68.0	65.1	66.0	68.6	66.4
**Gujarat**	1981	55.2	61.2	60.3	58.8	65.4	63.3
	2009	66.0	68.1	64.9	69.9	72.4	69.5
**Haryana**	1981	58.9	63.8	61.7	58.4	64.3	63.7
	2009	65.1	67.3	64.0	70.1	73.0	69.9
**Himachal Pradesh**	1983	60.1	64.5	62.6	62.0	65.9	64.5
	2009	67.5	69.4	65.7	72.1	74.5	71.1
**Karnataka**	1981	58.5	62.3	60.8	61.2	64.7	64.2
	2009	65.2	66.9	63.4	70.0	72.1	68.6
**Kerala**	1981	64.7	66.4	63.8	69.6	71.0	68.3
	2009	71.4	71.2	67.4	77.2	77.2	73.4
**Madhya Pradesh**	1981	50.3	57.5	58.6	50.2	56.9	59.8
	2009	61.7	65.0	62.2	64.8	68.5	66.2
**Maharashtra**	1981	59.0	63.0	61.1	60.0	64.0	63.1
	2009	68.1	69.2	65.6	72.1	73.4	69.9
**Odisha**	1981	52.6	60.0	58.9	51.5	58.5	58.5
	2009	62.9	66.2	63.5	64.4	67.9	65.3
**Punjab**	1981	63.8	68.5	66.0	66.7	71.9	71.4
	2009	67.6	69.1	65.6	72.0	74.0	70.8
**Rajasthan**	1981	53.6	58.9	59.3	53.9	59.2	61.4
	2009	65.4	68.3	65.1	69.3	72.7	70.4
**Tamil Nadu**	1981	55.4	60.1	59.2	55.9	60.3	60.1
	2009	67.6	68.5	64.8	71.4	72.5	68.8
**Uttar Pradesh**	1981	51.0	58.8	58.9	48.0	56.2	58.9
	2009	62.2	65.3	62.4	64.5	68.0	66.1
**West Bengal**	1983	56.5	60.8	60.0	57.9	61.9	61.7
	2009	67.7	69.0	65.5	71.0	72.5	69.0
