# Supplementary material for: Threshold Levels of Infant and Under-Five Mortality for Crossover between Life Expectancies at Ages Zero, One and Five in India: A Decomposition Analysis
Source: PLoS One. 2015 Dec 18;10(12):e0143764. doi: 10.1371/journal.pone.0143764 (PMC4684288; doi:10.1371/journal.pone.0143764)
Supplement: S2 Table — (PDF) [file pone.0143764.s007.pdf]

S2A Table: Levels of crossover between life expectancies at ages zero and five and corresponding $\left({}_{1}q_{0}\right)$, under-five $\left({}_{0}q_{5}\right)$ and adult mortality $\left({}_{45}q_{15}\right)$, various model life tables

	Male						Female					
	e_0^0	e_5^0	$e_0^0 - e_5^0$	${}_{1}q_{0}$	${}_{0}q_{5}$	${}_{45}q_{15}$	e_0^0	e_5^0	$e_0^0 - e_5^0$	${}_{1}q_{0}$	${}_{0}q_{5}$	${}_{45}q_{15}$
**Coale and Demeny model life tables**												
**East**	64.0	63.8	0.25	0.06	0.07	0.22	67.0	66.8	0.22	0.06	0.07	0.16
**North**	64.0	63.8	0.23	0.05	0.07	0.22	66.0	65.8	0.20	0.05	0.07	0.19
**South**	69.0	68.7	0.28	0.05	0.07	0.13	72.0	71.8	0.19	0.05	0.06	0.09
**West**	63.0	62.7	0.26	0.06	0.07	0.24	65.0	64.8	0.25	0.05	0.07	0.21
**United Nations model life tables**												
**Chilean**	65.0	64.9	0.10	0.06	0.07	0.22	70.0	69.9	0.07	0.06	0.07	0.14
**Far East Asian**	57.0	56.9	0.11	0.06	0.08	0.37	62.0	61.8	0.22	0.05	0.07	0.28
**General**	64.0	63.9	0.08	0.06	0.07	0.23	67.0	67.0	0.04	0.05	0.07	0.18
**Latin**	67.0	66.9	0.08	0.05	0.07	0.18	70.0	69.8	0.16	0.05	0.07	0.14
**South Asian**	69.0	68.8	0.22	0.05	0.07	0.14	72.0	71.7	0.30	0.05	0.06	0.09

S2B Table: Levels of crossover between life expectancies at ages zero and one and corresponding infant $\left({}_{1}q_{0}\right)$, under-five $\left({}_{0}q_{5}\right)$ and adult mortality $\left({}_{45}q_{15}\right)$, various model life tables

	Male						Female					
	$\mathbf{e}_0^0$	$\mathbf{e}_1^0$	$e_0^0 - e_1^0$	${}_{1}q_{0}$	${}_{0}q_{5}$	${}_{45}q_{15}$	$\mathbf{e}_0^0$	$\mathbf{e}_1^0$	$e_0^0 - e_1^0$	${}_{1}q_{0}$	${}_{0}q_{5}$	${}_{45}q_{15}$
**Coale and Demeny model life tables**												
**East**	75.0	75.0	0.04	0.01	0.01	0.10	81.0	81.0	0.05	0.01	0.01	0.05
**North**	79.0	78.9	0.06	0.01	0.01	0.08	81.0	81.0	0.03	0.01	0.02	0.06
**South**	84.0	84.0	0.05	0.01	0.01	0.03	87.0	86.9	0.07	0.01	0.01	0.02
**West**	75.0	74.9	0.06	0.01	0.01	0.10	78.0	78.0	0.03	0.01	0.02	0.07
**United Nations model life tables**												
**Chilean**	82.0	82.0	0.01	0.01	0.01	0.05	88.0	87.9	0.07	0.01	0.01	0.03
**Far East Asian**	74.0	73.9	0.09	0.01	0.01	0.11	82.0	82.0	0.04	0.01	0.01	0.06
**General**	81.0	80.9	0.07	0.01	0.01	0.06	85.0	85.0	0.03	0.01	0.01	0.03
**Latin**	82.0	82.0	0.00	0.01	0.02	0.05	85.0	85.0	0.02	0.01	0.02	0.04
**South Asian**	83.0	83.0	0.01	0.01	0.02	0.04	86.0	86.0	0.03	0.01	0.01	0.02
