# Supplementary material for: Threshold Levels of Infant and Under-Five Mortality for Crossover between Life Expectancies at Ages Zero, One and Five in India: A Decomposition Analysis
Source: PLoS One. 2015 Dec 18;10(12):e0143764. doi: 10.1371/journal.pone.0143764 (PMC4684288; doi:10.1371/journal.pone.0143764)
Supplement: S3 Table — Arranged in descending order of “Below age one” component in the period 2005–09. (PDF) [file pone.0143764.s008.pdf]

S3 Table: Components (in %) of change in gap between life expectancies at ages zero and one, India and States, 1981-09

India/States	1981-85 <1 / ≥1	1985-89 <1 / ≥1	1989-93 <1 / ≥1	1993-97 <1 / ≥1	1997-01 <1 / ≥1	2001-05 <1 / ≥1	2005-09 <1 / ≥1
	**Relative share: Men**						
**India**	**113.4 / -13.4**	**121.3 / -21.3**	**119.0 / -19.0**	**120.3 / -20.3**	**120.4 / -20.4**	**112.7 / -12.7**	**104.6 / -4.6**
**Gujarat**	111.3 / -11.3	99.5 / 0.5	118.4 / -18.4	230.9 / -130.9	111.4 / -11.4	109.7 / -9.7	116.6 / -16.6
**Orissa**	118.8 / -18.8	156.4 / -56.4	113.2 / -13.2	107.3 / -7.3	111.7 / -11.7	111.2 / -11.2	115.5 / -15.5
**Punjab**	118.8 / -18.8	156.4 / -56.4	113.2 / -13.2	107.3 / -7.3	111.7 / -11.7	111.2 / -11.2	115.5 / -15.5
**Madhya Pradesh**	134.3 / -34.3	114.5 / -14.5	123.6 / -23.6	126.9 / -26.9	107.5 / -7.5	125.4 / -25.4	109.4 / -9.4
**Bihar**	- / -	148.8 / -48.8	120.5 / -20.5	151.1 / -51.1	167.2 / -67.2	50.7 / 49.3	107.9 / -7.9
**Rajasthan**	62.1 / 37.9	111.7 / -11.7	220.6 / -120.6	2266.7 / -2166.7	165.6 / -65.6	111.1 / -11.1	105.8 / -5.8
**Maharashtra**	107.9 / -7.9	124.8 / -24.8	116.3 / -16.3	101.5 / -1.5	110.8 / -10.8	110.7 / -10.7	104.5 / -4.5
**West Bengal**	- / -	158.3 / -58.3	108.6 / -8.6	123.8 / -23.8	136.3 / -36.3	103.8 / -3.8	104.2 / -4.2
**Assam**	12.9 / 87.1	134.1 / -34.1	132.3 / -32.3	106.3 / -6.3	123.7 / -23.7	-303.6 / 403.6	102.5 / -2.5
**Kerala**	106.8 / -6.8	103.8 / -3.8	109.4 / -9.4	92.7 / 7.3	101.6 / -1.6	62.1 / 37.9	102.4 / -2.4
**Tamil Nadu**	112.9 / -12.9	119.7 / -19.7	118.4 / -18.4	108.0 / -8.0	147.0 / -47.0	110.8 / -10.8	102.2 / -2.2
**Uttar Pradesh**	100.9 / -0.9	112.7 / -12.7	112.2 / -12.2	169.7 / -69.7	133.1 / -33.1	108.6 / -8.6	101.3 / -1.3
**Karnataka**	37.3 / 62.7	72.8 / 27.2	107.3 / -7.3	107.8 / -7.8	64.3 / 35.7	105.5 / -5.5	99.8 / 0.2
**Andhra Pradesh**	134.9 / -34.9	83.0 / 17.0	129.0 / -29.0	117.8 / -17.8	187.8 / -87.8	112.7 / -12.7	98.2 / 1.8
**Himachal Pradesh**	- / -	109.0 / -9.0	84.8 / 15.2	160.0 / -60.0	138.9 / -38.9	100.1 / -0.1	93.5 / 6.5
**Haryana**	142.1 / -42.1	100.5 / -0.5	113.3 / -13.3	253.7 / -153.7	93.2 / 6.8	109.7 / -9.7	89.9 / 10.1
	**Relative share: Women**						
**India**	**116.2 / -16.2**	**129.9 / -29.9**	**131.3 / -31.3**	**171.3 / -71.3**	**150.8 / -50.8**	**119.9 / -19.9**	**112.5 / -12.5**
**Assam**	165.8 / -65.8	133.5 / -33.5	128.0 / -28.0	-1142.9 / 1242.9	26.2 / 73.8	117.9 / -17.9	338.3 / -238.3
**Madhya Pradesh**	120.7 / -20.7	-69.5 / 169.5	136.4 / -36.4	142.7 / -42.7	268.3 / -168.3	117.2 / -17.2	124.2 / -24.2
**Kerala**	120.5 / -20.5	101.7 / -1.7	107.8 / -7.8	134.8 / -34.8	107.8 / -7.8	86.9 / 13.1	123.1 / -23.1
**Uttar Pradesh**	104.5 / -4.5	130.6 / -30.6	135.9 / -35.9	128.3 / -28.3	159.7 / -59.7	129.0 / -29.0	114.3 / -14.3
**Bihar**	- / -	133.6 / -33.6	121.4 / -21.4	205.5 / -105.5	639.6 / -539.6	158.5 / -58.5	110.7 / -10.7
**Orissa**	110.6 / -10.6	-552.2 / 652.2	123.5 / -23.5	146.6 / -46.6	135.7 / -35.7	120.6 / -20.6	110.4 / -10.4
**Punjab**	82.4 / 17.6	101.2 / -1.2	196.2 / -96.2	695.2 / -595.2	161.9 / -61.9	114.1 / -14.1	101.1 / -1.1
**West Bengal**	- / -	110.5 / -10.5	466.1 / -366.1	112.3 / -12.3	148.1 / -48.1	115.3 / -15.3	108.4 / -8.4
**Gujarat**	134.7 / -34.7	86.1 / 13.9	116.3 / -16.3	-20.5 / 120.5	-80.8 / 180.8	109.6 / -9.6	107.6 / -7.6
**Tamil Nadu**	179.0 / -79.0	124.3 / -24.3	128.6 / -28.6	306.9 / -206.9	121.4 / -21.4	108.5 / -8.5	106.7 / -6.7
**Rajasthan**	86.1 / 13.9	123.1 / -23.1	289.6 / -189.6	47.5 / 52.5	187.1 / -87.1	110.8 / -10.8	106.1 / -6.1
**Karnataka**	24.5 / 75.5	49.1 / 50.9	147.0 / -47.0	102.9 / -2.9	677.8 / -577.8	106.8 / -6.8	103.6 / -3.6
**Maharashtra**	128.6 / -28.6	113.8 / -13.8	128.4 / -28.4	132.1 / -32.1	322.6 / -222.6	116.0 / -16.0	103.0 / -3.0
**Andhra Pradesh**	131.6 / -31.6	117.4 / -17.4	126.3 / -26.3	26.2 / 73.8	136.3 / -36.3	128.9 / -28.9	98.5 / 1.5
**Haryana**	135.3 / -35.3	118.7 / -18.7	104.1 / -4.1	44.0 / 56.0	-370.5 / 470.5	117.2 / -17.2	97.2 / 2.8
**Himachal Pradesh**	- / -	-214.1 / 314.1	180.1 / -80.1	-9.0 / 109.0	111.2 / -11.2	74.4 / 25.6	82.2 / 17.8

Note: Arranged in descending order of “Below age one” component in the period 2005-09.
