# Supplementary material for: Threshold Levels of Infant and Under-Five Mortality for Crossover between Life Expectancies at Ages Zero, One and Five in India: A Decomposition Analysis
Source: PLoS One. 2015 Dec 18;10(12):e0143764. doi: 10.1371/journal.pone.0143764 (PMC4684288; doi:10.1371/journal.pone.0143764)
Supplement: S4 Table — Arranged in descending order of “Below age five” component in the period 2005–09. (PDF) [file pone.0143764.s009.pdf]

S4 Table: Components (in %) of change in gap between life expectancies at ages zero and five, India and States, 1981-09

India/States	1981-85 <5 / ≥5	1985-89 <5 / ≥5	1989-93 <5 / ≥5	1993-97 <5 / ≥5	1997-01 <5 / ≥5	2001-05 <5 / ≥5	2005-09 <5 / ≥5
	**Relative share: Men**						
**India**	**107.1 / -7.1**	**108.9 / -8.9**	**106.9 / -6.9**	**104.7 / -4.7**	**110.8 / -10.8**	**110.9 / -10.9**	**101.5 / -1.5**
**Orissa**	113.0 / -13.0	201.5 / -101.5	106.3 / -6.3	98.5 / 1.5	112.0 / -12.0	108.6 / -8.6	115.0 / -15.0
**Gujarat**	106.0 / -6.0	95.6 / 4.4	112.3 / -12.3	119.6 / -19.6	104.7 / -4.7	107.8 / -7.8	110.6 / -10.6
**Maharashtra**	104.2 / -4.2	108.6 / -8.6	107.1 / -7.1	98.6 / 1.4	106.7 / -6.7	110.1 / -10.1	107.1 / -7.1
**Bihar**	- / -	123.2 / -23.2	108.5 / -8.5	97.1 / 2.9	130.2 / -30.2	50.5 / 49.5	105.4 / -5.4
**West Bengal**	- / -	109.5 / -9.5	102.3 / -2.3	104.9 / -4.9	117.1 / -17.1	102.8 / -2.8	104.2 / -4.2
**Madhya Pradesh**	122.7 / -22.7	99.8 / 0.2	99.9 / 0.1	104.9 / -4.9	103.9 / -3.9	121.7 / -21.7	103.3 / -3.3
**Tamil Nadu**	107.4 / -7.4	104.6 / -4.6	105.3 / -5.3	102.0 / -2.0	138.8 / -38.8	110.1 / -10.1	101.7 / -1.7
**Rajasthan**	58.6 / 41.4	101.6 / -1.6	110.7 / -10.7	159.2 / -59.2	115.9 / -15.9	109.1 / -9.1	101.6 / -1.6
**Kerala**	105.0 / -5.0	100.7 / -0.7	107.4 / -7.4	90.2 / 9.8	100.0 / 0.0	63.6 / 36.4	100.8 / -0.8
**Assam**	11.3/ 88.7	110.7 / -10.7	146.5/ -46.5	95.8/ 4.2	106.4/ -6.4	-308.1/ 408.1	99.4 / 0.6
**Karnataka**	35.9 / 64.1	122.3 / -22.3	98.3 / 1.7	104.3 / -4.3	138.2 / -38.2	104.5 / -4.5	99.0 / 1.0
**Uttar Pradesh**	94.4 / 5.6	111.6 / -11.6	104.4 / -4.4	122.5 / -22.5	105.7 / -5.7	106.0 / -6.0	97.5 / 2.5
**Andhra Pradesh**	129.2 / -29.2	87.7 / 12.3	112.4 / -12.4	105.9 / -5.9	106.1 / -6.1	111.9 / -11.9	97.4 / 2.6
**Punjab**	92.9 / 7.1	102.9 / -2.9	104.2 / -4.2	79.5 / 20.5	113.7 / -13.7	110.6 / -10.6	94.4 / 5.6
**Himachal Pradesh**	- / -	99.9 / 0.1	88.5 / 11.5	133.8 / -33.8	131.1 / -31.1	99.6 / 0.4	92.9 / 7.1
**Haryana**	137.8 / -37.8	97.6 / 2.4	116.1 / -16.1	136.2 / -36.2	92.1 / 7.9	108.8 / -8.8	88.3 / 11.7
	**Relative share: Women**						
**India**	**107.2 / -7.2**	**114.3 / -14.3**	**109.9 / -9.9**	**115.2 / -15.2**	**112.5 / -12.5**	**117.2 / -17.2**	**108.5 / -8.5**
**Madhya Pradesh**	106.9 / -6.9	116.9 / -16.9	104.2 / -4.2	109.9 / -9.9	128.4 / -28.4	112.9 / -12.9	111.9 / -11.9
**Rajasthan**	77.6 / 22.4	104.0 / -4.0	114.7 / -14.7	0.0 / 100.0	110.3 / -10.3	108.2 / -8.2	111.0 / -11.0
**Bihar**	- / -	134.2 / -34.2	108.7 / -8.7	104.3 / -4.3	126.8 / -26.8	154.7 / -54.7	110.4 / -10.4
**Orissa**	103.6 / -3.6	-0.4 / 100.4	147.5 / -47.5	103.9 / -3.9	120.5 / -20.5	117.7 / -17.7	108.2 / -8.2
**Uttar Pradesh**	93.0 / 7.0	127.5 / -27.5	114.1 / -14.1	107.4 / -7.4	106.5 / -6.5	123.6 / -23.6	108.0 / -8.0
**Kerala**	118.6 / -18.6	99.7 / 0.3	105.2 / -5.2	117.5 / -17.5	103.1 / -3.1	86.4 / 13.6	107.0 / -7.0
**Tamil Nadu**	167.1 / -67.1	105.2 / -5.2	109.6 / -9.6	106.6 / -6.6	123.2 / -23.2	107.8 / -7.8	105.3 / -5.3
**Gujarat**	131.0 / -31.0	60.3 / 39.7	111.0 / -11.0	203.6 / -103.6	120.2 / -20.2	107.5 / -7.5	104.1 / -4.1
**West Bengal**	- / -	96.1 / 3.9	!42.5 / -42.5	105.8 / -5.8	115.6 / -15.6	113.9 / -13.9	102.9 / -2.9
**Maharashtra**	122.8 / -22.8	105.4 / -5.4	103.0 / -3.0	111.7 / -11.7	121.9 / -21.9	114.7 / -14.7	102.6 / -2.6
**Karnataka**	20.4 / 79.6	109.6 / -9.6	103.0 / -3.0	99.7 / 0.3	132.9 / -32.9	105.5 / -5.5	100.9 / -0.9
**Punjab**	78.8 / 21.2	98.1 / 1.9	106.5 / -6.5	147.6 / -47.6	-5.7 / 105.7	112.5 / -12.5	100.2 / -0.2
**Andhra Pradesh**	125.5 / -25.5	103.2 / -3.2	104.7 / -4.7	300.9 / -200.9	106.9 / -6.9	128.2 / -28.2	96.9 / 3.1
**Haryana**	128.3 / -28.3	116.3 / -16.3	103.5 / -3.5	-248.1 / 348.1	128.3 / -28.3	114.9 / -14.9	94.9 / 5.1
**Himachal Pradesh**	- / -	131.8 / -31.8	152.7 /-52.7	-27.2 / 127.2	108.1 / -8.1	73.8 / 26.2	84.3 / 15.7
**Assam**	153.9/ -53.9	118.4/ -18.4	121.7/ -21.7	104.7/ -4.7	137.4/ -37.4	114.9/ -14.9	-66.0 / 166

Note: Arranged in descending order of “Below age five” component in the period 2005-09.
